# Atypical presentations of fetal polycystic kidney disease demonstrates the utility of a genomic autopsy for accurate post-mortem diagnoses

**DOI:** 10.1186/s40246-025-00844-4

**Published:** 2025-11-12

**Authors:** Mahalia S. B. Frank, Melissa K. Bennett, Thuong T. Ha, Lynette Moore, Peer Arts, Alicia B. Byrne, Milena Babic, Luis Arriola-Martinez, John Toubia, Peter J. Brautigan, Jinghua Feng, Quenten Schwarz, Paul Q. Thomas, Sandra G. Piltz, Melissa A. White, Ali Moghimi, Kate Strachan, Edward Kwan, Amanda Springer, Miranda Lewit-Mendes, Jarrad Dearman, Tenielle Davis, Lucy Kevin, Hugh J. McCarthy, Jan Liebelt, Emma Krzesinski, Matthew Regan, Kunal Verma, George McGillivray, Kushani Jayasinghe, Matilda R. Jackson, Christopher P. Barnett, Hamish S. Scott

**Affiliations:** 1 Department of Genetics and Molecular Pathology, Centre for Cancer Biology, an alliance between SA Pathology and the University of South Australia, Frome Road, Adelaide, SA 5000 Australia; 2 ACRF Genomics Facility, Centre for Cancer Biology, an alliance between SA Pathology and the University of South Australia, Adelaide, SA Australia; 3 Adelaide Medical School, University of Adelaide, Adelaide, SA 5000 Australia; 4 UniSA Clinical and Health Sciences, University of South Australia, Adelaide, SA Australia; 5 Department of Anatomical Pathology, SA Pathology, Women’s and Children’s Hospital, North Adelaide, SA Australia; 6 Robinson Research Institute, School of Biomedicine, The University of Adelaide, Adelaide, SA Australia; 7 South Australian Health and Medical Research Institute, Adelaide, SA Australia; 8 The Children’s Hospital at Westmead Clinical School, Faculty of Medicine and Health, University of Sydney, Sydney, NSW Australia; 9 Department of Histopathology, The Children’s Hospital at Westmead, Sydney, NSW Australia; 10 Anatomical Pathology, Royal Women’s Hospital (A Division of Royal Children’s Hospital Laboratory Services), Melbourne, VIC Australia; 11 Department of Pathology, Monash Health, Melbourne, VIC Australia; 12 Department of Paediatrics, Monash University, Melbourne, VIC Australia; 13 Australian Institute of Health Innovation, Faculty of Medicine, Health and Human Sciences, Macquarie University, Sydney, Australia; 14 Monash Genetics, Monash Health, Melbourne, VIC Australia; 15 Department of Paediatrics, University of Melbourne, Melbourne, VIC Australia; 16 Paediatric and Reproductive Genetics Unit, South Australian Clinical Genetics Service, Women’s and Children’s Hospital, North Adelaide, SA Australia; 17 Royal Women’s Hospital, Melbourne, VIC Australia; 18 Department of Clinical Genetics, Sydney Children’s Hospitals Network-Westmead, Sydney, NSW Australia; 19 KidGen Collaborative, Australian Genomics Health Alliance, Parkville, VIC Australia; 20 Discipline of Child and Adolescent Health, Faculty of Medicine and Health, The University of Sydney, Sydney, NSW Australia; 21 Centre for Kidney Research, Kids Research Institute and the Department of Nephrology, The Children’s Hospital at Westmead, Westmead, NSW Australia; 22 Victorian Clinical Genetics Services, Murdoch Children’s Research Institute and Royal Women’s Hospital, Melbourne, VIC Australia; 23 Department of Nephrology, Monash Health, Clayton, VIC Australia; 24 Department of Medicine, Monash University, Melbourne, VIC Australia; 25 Australian Genomics, Parkville, VIC Australia

**Keywords:** Genomics, Prenatal, Pregnancy loss, Polycystic kidney disease, ADPKD, ARPKD

## Abstract

**Background:**

Prenatal presentation of polycystic kidney disease (PKD), characterized by bilateral renal cysts and enlarged echogenic kidneys on ultrasound, often results in perinatal death. Prenatal manifestations of PKD are generally associated with autosomal recessive PKD, most commonly a result of pathogenic variants in *PKHD1*, but in rare cases can also be driven by bi-allelic inheritance of pathogenic variants in genes more commonly associated with autosomal dominant PKD such as *PKD1*. Diagnosing the underlying cause of prenatal PKD can be complicated by atypical histology, and/or a prenatal phenotype that does not align with family history. In this study, five cases of prenatal PKD with atypical or inconclusive features identified during post-mortem investigations underwent trio exome or genome sequencing, termed a genomic autopsy.

**Results:**

Genomic autopsy was able to delineate the genetic basis of prenatal PKD in all five families.

**Conclusion:**

Our findings demonstrate the diagnostic utility of a genomic autopsy in providing a genetic diagnosis for fetal PKD cases post-mortem, particularly in atypical presentations. A genetic diagnosis is highly beneficial for future family planning, including the use of reproductive technologies, as well as identifying presymptomatic parents who are likely to develop PKD in the future.

**Supplementary Information:**

The online version contains supplementary material available at 10.1186/s40246-025-00844-4.

## Background

Polycystic kidney disease (PKD) is an inherited condition that causes cysts to form in the kidneys ± liver, eventually leading to organ failure. PKD has broadly been classified according to inheritance patterns, with autosomal dominant PKD (ADPKD) being defined as a late-onset disease and autosomal recessive PKD (ARPKD) being characterized as a more severe, early-onset disease [1–4].

The prevalence of ADPKD is estimated between 1:1000 and 1:2500, and is characterized by large, rounded renal cysts developing in the medulla and cortex in proximal tubules on histopathological assessment, often with glomerular involvement [3, 4]. Common extra-renal phenotypes in ADPKD include hepatic and pancreatic cysts (prevalence of which increases with age), cardiovascular disorders, and abdominal wall hernias [4]. End-stage renal disease occurs in approximately 50% of patients by 60 years of age [3, 4].

ADPKD is most commonly associated with genetic variants in the polycystin-1 and 2 genes, *PKD1* (OMIM 601313) and *PKD2* (OMIM 173910)*,* that are core components of primary cilia in renal tubular cells [1]. Both genes are core regulators of cellular processes in the kidney such as fluid transport, differentiation, proliferation, apoptosis and cell adhesion. A decrease in polycystin-1 or 2 expression below a critical threshold causes the impairment of several intracellular signaling pathways, terminating in the development of cysts due to cell proliferation and fluid secretion [5, 6]. Over 2000 pathogenic (P) or likely pathogenic (LP) variants in *PKD1* and over 400 P/LP variants in *PKD2* have been reported to date in ClinVar [7], most of which are frameshift or nonsense variants, though missense variants have also been reported. Though *PKD1* and *PKD2* variants are by far the most common variants found in ADPKD patients (~ 67% and ~ 15% respectively) [8], other, less common, causative genes are now also recognized as causing ADPKD [8, 9], as listed in Table 1. Many of these genes, including intraflagellar transport 140 (*IFT140*, OMIM 614620), DnaJ heat shock protein family (HSP40) member B11 (*DNAJB11*, OMIM 611341), and glucosidase II alpha subunit (*GANAB*, OMIM 104160), encode proteins important for the synthesis, maturation, and translocation of the polycystin proteins through the endoplasmic reticulum [10–13]. However, pathogenic variants in these less common genes typically manifest with differing levels of severity or atypical phenotypes compared to *PKD1* (Table 1).

**Table 1:** Phenotypes, presentations, and associated genes for polycystic kidney disease.

Disease	Common phenotypes	Common histology	Gene	ClinGen Classification	Gene-specific findings [9, 10, 19, 22, 35–37]
Autosomal dominant polycystic kidney disease	Multiple bilateral kidney cysts, usually manifesting in adulthood. Severe disease may present as large, echogenic kidneys in infants/children Extra-renal phenotypes can include hepatic cysts (prevalence increases with age), pancreatic cysts, cardiovascular disorders (e.g. mitral valve and/or aortic regurgitation, aortic dilation and dissection, aneurysm), and abdominal wall hernias	Rounded, large cysts developing in medulla or cortes in renal tubes or collecting tubes, glomerular cysts sometimes present	*PKD1*	Definitive	
			*PKD2*	Definitive	Later onset compared to *PKD1* variants
			*DNAJB11*	Definitive	Later onset. Cysts generally remain small, so kidneys are not always enlarged, but commonly develop interstitial fibrosis—shares some phenotypic overlap with autosomal dominant tubulointerstitial kidney disease. Cardiovascular phenotypes may also be present
			*GANAB*	Definitive	Majority of individuals develop liver cysts
			*IFT140*	Definitive	Often milder phenotype compared to *PKD1*
			*ALG8*	Definitive	Mild phenotype compared to *PKD1* variants
			*ALG9*	Definitive	Generally mild to moderate phenotype compared to *PKD1* variants
			*NEK8*	Strong	Often early-onset, may be detected prenatally or in infants
			*ALG5*	Moderate	Kidneys commonly non-enlarged but show interstitial fibrosis
			*ALG6*	Limited	Mild phenotype compared to *PKD1* variants
Autosomal recessive polycystic kidney disease	Usually detected prenatally or in infants. Bilateral and symmetrically enlarged kidneys with hyperechogenicity. If detected prenatally, oligohydramnios/anhydramnios may be present Ductal malformation may also be present	Fusiform cystic dilation of the collecting ducts into medulla and extending towards the cortex, normal glomeruli. Single or multiple small elongated cysts sometimes present	*PKHD1*	Definitive	
			*PKD1*	Definitive	Histological findings may be more in line with ADPKD, with glomerular cysts present
			*DZIP1L*	Definitive	Less evidence for presence of ductal plate malformation
			*PMM2*	Moderate	Currently known to be caused by a specific promoter variant (c.-167G > T)
			*DNAJB11*	Limited	May also present with liver and pancreatic legions/fibrosis typical of ciliopathies. Cardiovascular phenotypes may also be present
			*CYS1*	Limited	

The genes currently listed on ClinGen for autosomal dominant polycystic kidney disease and autosomal recessive polycystic kidney disease, including common phenotypes and histological presentation, current ClinGen classification, and additional gene-specific findings.

In contrast to ADPKD, the prevalence of ARPKD is around 1 in 20,000 [14]. It is often identified during pregnancy and manifests as symmetrical bilateral enlarged echo-dense kidneys with little to no amniotic fluid on ultrasound [15]. It is distinguishable from ADPKD on histopathological assessment as cystic dilatation occurs in the medullary collecting ducts with normal glomeruli. There is often a loss of corticomedullary differentiation, and ductal plate malformations may be present [15, 16]. A lack of fluid production during the second half of a pregnancy can subsequently affect the development of the lungs and approximately 1 in 3 neonates with ARPKD die due to pulmonary complications [14]. For patients who survive the early post-natal period, end-stage renal disease occurs in approximately 60% of patients by 20 years of age [14, 15].

ARPKD is commonly attributed to genetic variants in the fibrocystin gene polycystic kidney and hepatic disease 1 (*PKHD1*, OMIM 606702) and the presence of at least one truncating variant is predictive of perinatal lethality [1]. A review in 2021 stated that approximately 750 *PKHD1* variants have been identified in ARPKD cases, around half of which are missense [15]. Studies have also shown that truncating mutations are present in about 55% of the most severe cases—medical pregnancy termination or early demise patients—but only in about 20% of live patients. Fibrocystin also localizes to the primary cilia, and experiments in *PKHD1*-silenced HEK-293 T cells suggest that the role of *PKHD1* in cyst formation is linked to modulation of intracellular calcium levels, promoting excessive epidermal growth factor-induced cellular proliferation [14]. Similar to ADPKD, there are genes other than *PKHD1* that have been linked to ARPKD (Table 1). DAZ interacting zinc finger protein 1 like (*DZIP1L*, OMIM617570) is an example of one such gene, with loss of the *DZIP1L* inhibiting the ciliary membrane translocation of *PKD1* and *PKD2* [17], resulting in ARPKD.

Though ADPKD and ARPKD have traditionally been thought of as separate conditions with their own genetic drivers, this is not always the case. In rare cases, biallelic inheritance in genes classically associated with ADPKD can results in a severe early onset phenotype that clinically resembles ARPKD. For example, inheriting two *PKD1* variants *in trans* can result in severe early onset PKD [18, 19], and, in one patient, the same phenotype was caused by a homozygous *PKD2* variant caused by uniparental disomy [20]. Indeed, ClinGen now lists *PKD1* as having a definitive gene-disease relationship with both ADPKD and ARPKD [9]. Likewise, whilst *DNAJB11* was initially discovered as a rare cause of ADPKD [10], it was later reported to cause a more severe phenotype when variants followed an autosomal recessive mode of inheritance [21, 22]. Reporting of these atypical early onset cases is important for advancing our understanding of prenatal phenotypes, as fetal presentations for Mendelian disorders remain poorly described compared to their post-natal counterparts [23, 24].

Here, we outline five cases of prenatal polycystic kidney disease from our larger Genomic Autopsy Study cohort [25] that presented atypically on post-mortem assessment. These families were offered a genomic autopsy—a post-mortem, unbiased genomic analysis following loss of a fetus or neonate (including termination for congenital abnormality), with the aim of determining the underlying genetic cause of the abnormality or death. For each of these five families, the genetic diagnosis of PKD provided by a genomic autopsy prompted further review of the post-mortem assessment due to an atypical presentation, and as such these cases are useful for increasing scientific knowledge. Here we demonstrate how a genomic autopsy approach can help inform pathological assessment and improve diagnoses of suspected fetal PKD, which can direct clinical counselling and reproductive care for families.

## Methods

### Cohort ascertainment

**Fig. 1:**
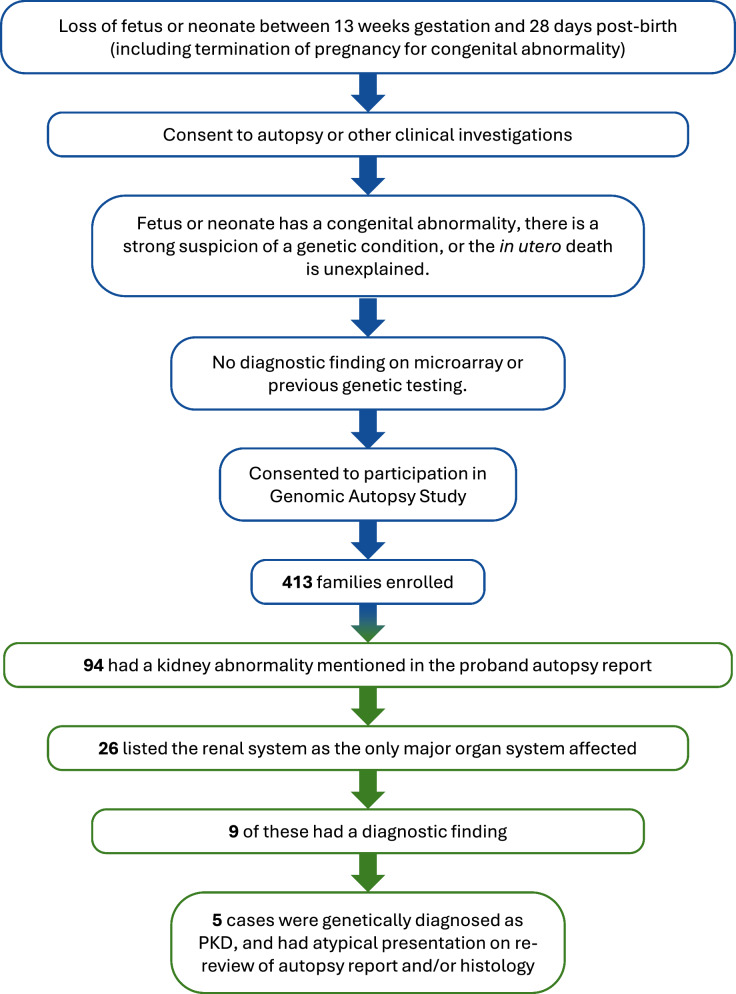
Cohort ascertainment and selection of cases for publication. The blue sections of the flowchart summarize how the Genomic Autopsy Study cohort was ascertained, and the green sections summarize how the five atypical PKD cases included here were selected for publication. When selecting the 26 cases where the renal system was the only major organ system affected, we did not exclude cases where non-renal phenotypes were likely linked to the renal abnormality (e.g. oligohydramnios). Cases were selected retrospectively, after non-biased genomic sequencing and analysis had occurred as part of the larger Genomic Autopsy Study [25]

This work was conducted as part of the Australian Genomic Autopsy Study [25]. Briefly, we performed parental-proband genomic sequencing for families who lost a fetus (including termination for congenital abnormality) or neonate between 13 weeks gestation and 28 days post-birth. To be included in the study, the proband was required to have had an autopsy or other standard-of-care clinical investigations, have either a congenital abnormality or have their death remain unexplained after investigations, and have no diagnostic findings on microarray or previous sequencing (Fig. 1). As part of the study, parents consented to the use of their, and their child’s, samples and clinical history, to allow detailed genetic and phenotypic information to be obtained.

### Tissue preparation and processing

Human kidney samples collected at autopsy and mouse kidney samples were embedded in paraffin, sectioned, and stained with haematoxylin and eosin as previously described [26]. Where the histological findings in the autopsy report did not align with the genetic finding, histology slides were re-analyzed to confirm.

### Nucleic acid isolation and next-generation sequencing

Parental DNA was isolated from whole blood or saliva. Fetal DNA was isolated from fetal tissue samples obtained at autopsy. DNA was extracted and sequenced on the Illumina NextSeq or NovaSeq Sequencing System as previously described [25].

### Bioinformatic analysis on NGS data

Sequences were aligned to the human reference genome (hg38) using Burrows-Wheeler Aligner (BWA-mem), and variants were annotated, prioritized and classified as described in [25]. More detail available in Supplementary Methods.

### TMEM212 functional follow-up

Details of methods utilized for functional follow-up of *TMEM212* are available in Supplementary Methods.

### RNA analysis for confirmation of splice effects

As previously described [25], reverse transcription PCR coupled with Nanopore sequencing was performed to aid the interpretation of *DNAJB11* variant effect in PED002.

## Results

On review of our Australian Genomic Autopsy Study, 94 of 413 enrolled cases had a kidney abnormality identified at autopsy. Among these, 26 only had major congenital abnormalities noted in the renal system (or associated phenotypes such as oligohydramnios), 13 of which noted polycystic kidneys as a phenotype. Genomic sequencing provided a diagnosis in only 9 of the 26 cases, including five families with an atypical presentation of PKD (Fig. 1). In all five families with atypical presentation of PKD, the proband had enlarged kidneys detected on fetal ultrasound prior to termination (four families) or neonatal death (one family), with two families having previously lost a pregnancy due to the same phenotype. Both parents and the proband were sequenced in all cases, with an affected sibling also sequenced if a DNA sample was available. Details of the clinical history and genetic finding for each family is given below.

### Case 1: PED002

**Fig. 2:**
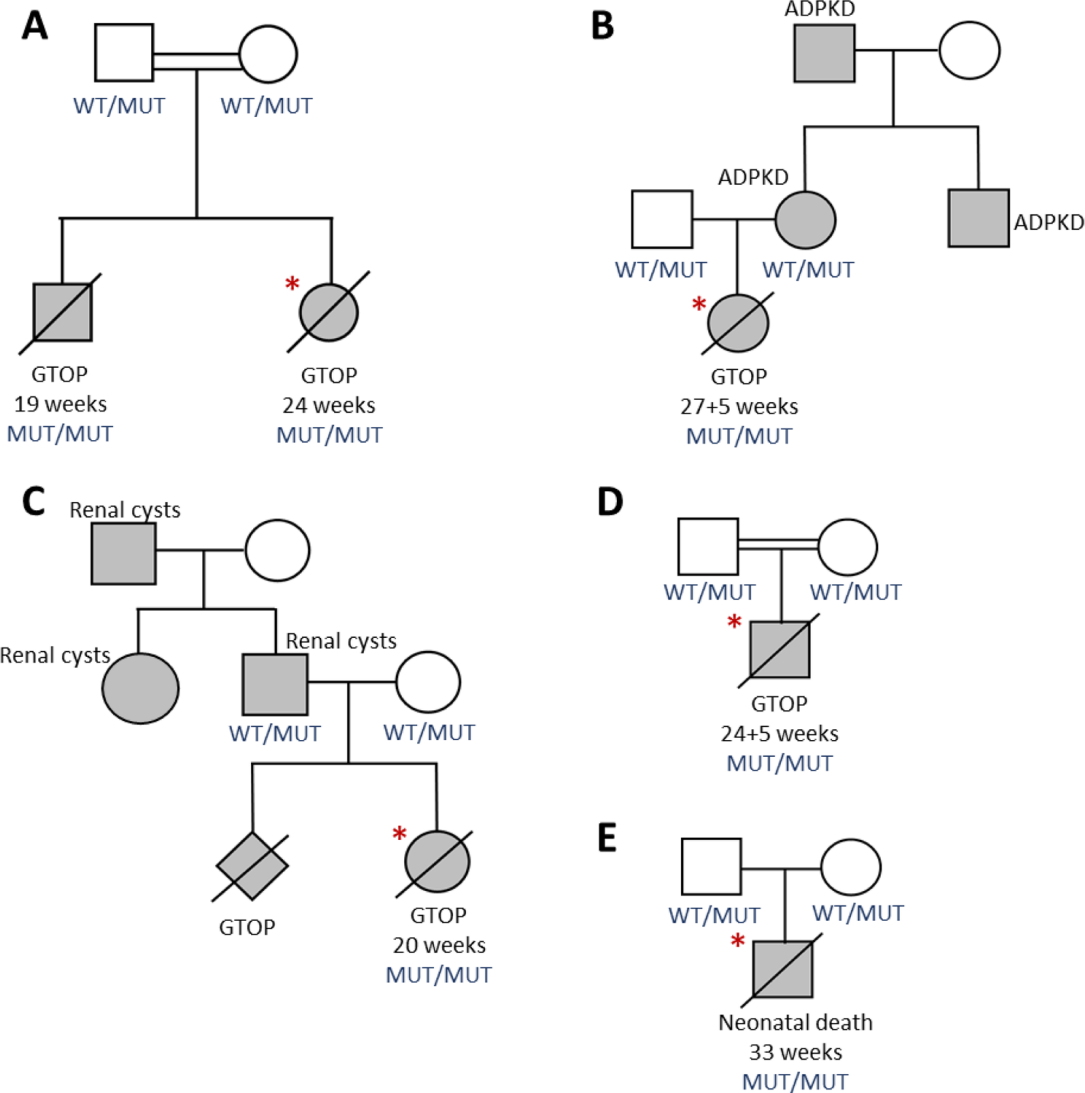
Pedigrees for each family showing individuals with polycystic kidneys. The proband is indicated by the red asterisks, sequenced individuals have their mutational status in the fetal PKD-causing gene shown in blue. **A** PED002, **B** PED283, **C** PED338, **D** PED138, **E** PED297. WT = wild-type allele, MUT = mutant allele, GTOP = gestational termination of pregnancy, shaded box indicates affected status

**Fig. 3:**
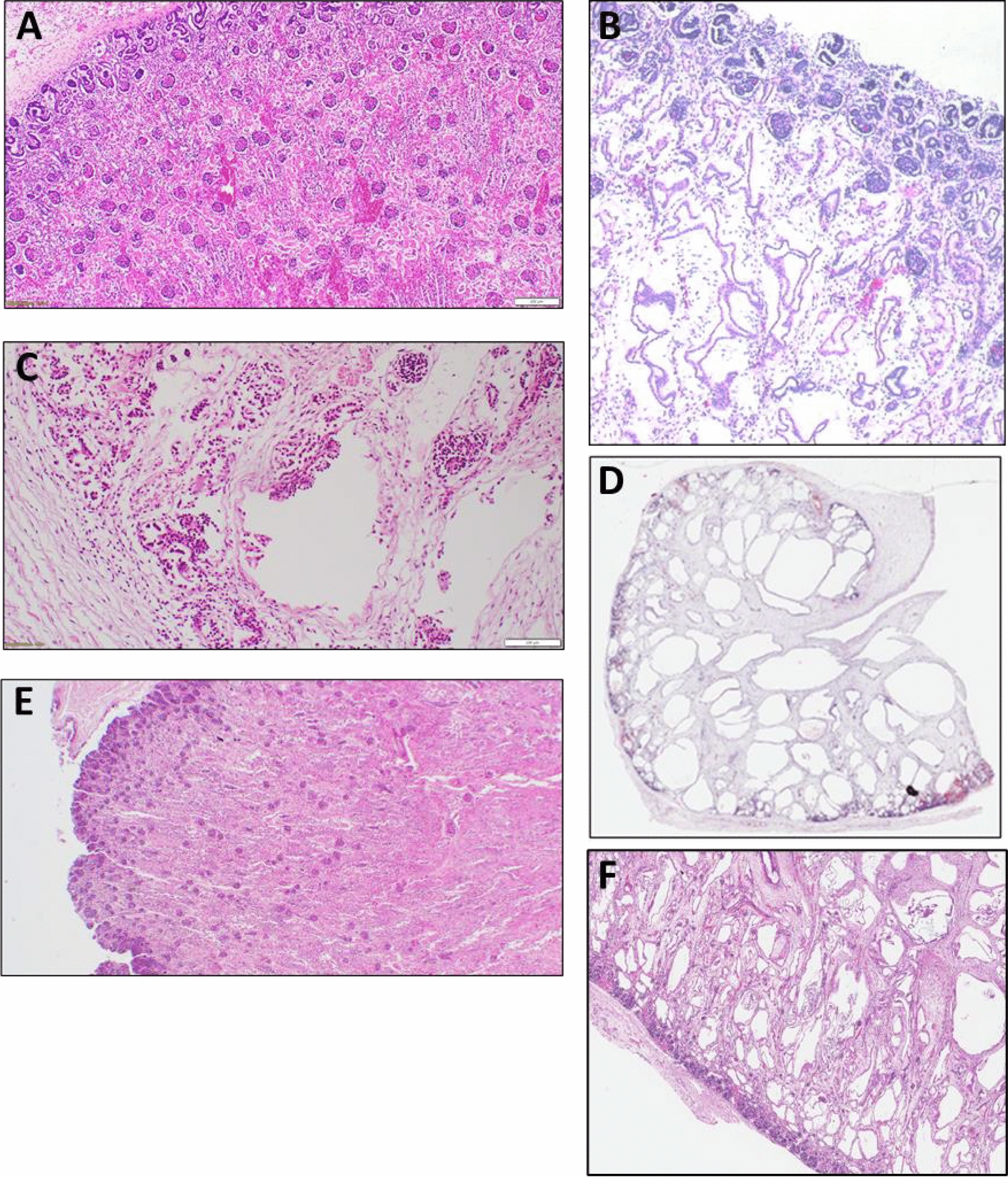
Kidney phenotypes were histologically assessed in each proband and reviewed after genomic analysis. Histological sections (haematoxylin and eosin stain) here are representative examples from each proband of the observed renal phenotypes. **A** Control section from a fetus with normal kidneys at 28 weeks gestation (magnified 25×). **B** PED002 (magnified 25×). **C** PED283 (magnified 63×). **D** PED338 (magnified 4×). **E** PED138 (magnified 20×). **F** PED297 (magnified 20×)

PED002 was a consanguineous couple who had two terminations of pregnancy (female at 24 weeks gestation and male at 19 weeks gestation) due to enlarged kidneys and severe oligohydramnios, suggestive of ARPKD (pedigree in Fig. 2A). Histopathological assessment of the kidneys in the female proband revealed non-cystic glomeruli, increased connective tissue in the deep cortex and medulla, and cystic dilation of the collecting ducts. These cysts were somewhat radial, but their irregular, jagged appearance was not completely typical of ARPKD (Fig. 3B), nor were the cysts typical of ADPKD or diffuse cystic dysplasia. It was noted that the findings were similar to those observed in the previous affected male sibling (histology not shown).

In both the proband and sibling, genomic sequencing did not initially identify any clinically significant variants that explained the recurrent phenotype. However, a run of homozygosity (ROH) that included four variants predicted to have a high impact on gene function was identified. Utilizing information available at the time of variant curation, biparental homozygous missense variants in poorly characterized transmembrane protein 212 (*TMEM212*, NM_001164436.1:c.331G > A) was deemed most likely to be the causative variant. However, further mouse model functional follow-up was conducted which challenged this hypothesis. Whilst *TMEM212* was found to be expressed in fetal kidney and some overlap was seen histopathologically between the kidney phenotype observed in the subsequently generated *Tmem212* knockout mouse model and the human histopathology, it was significantly less severe than in the patients and considered insufficient to account for the severity of the patient’s phenotype and clinical outcome (Supplementary Figure S4-6).

**Fig. 4:**
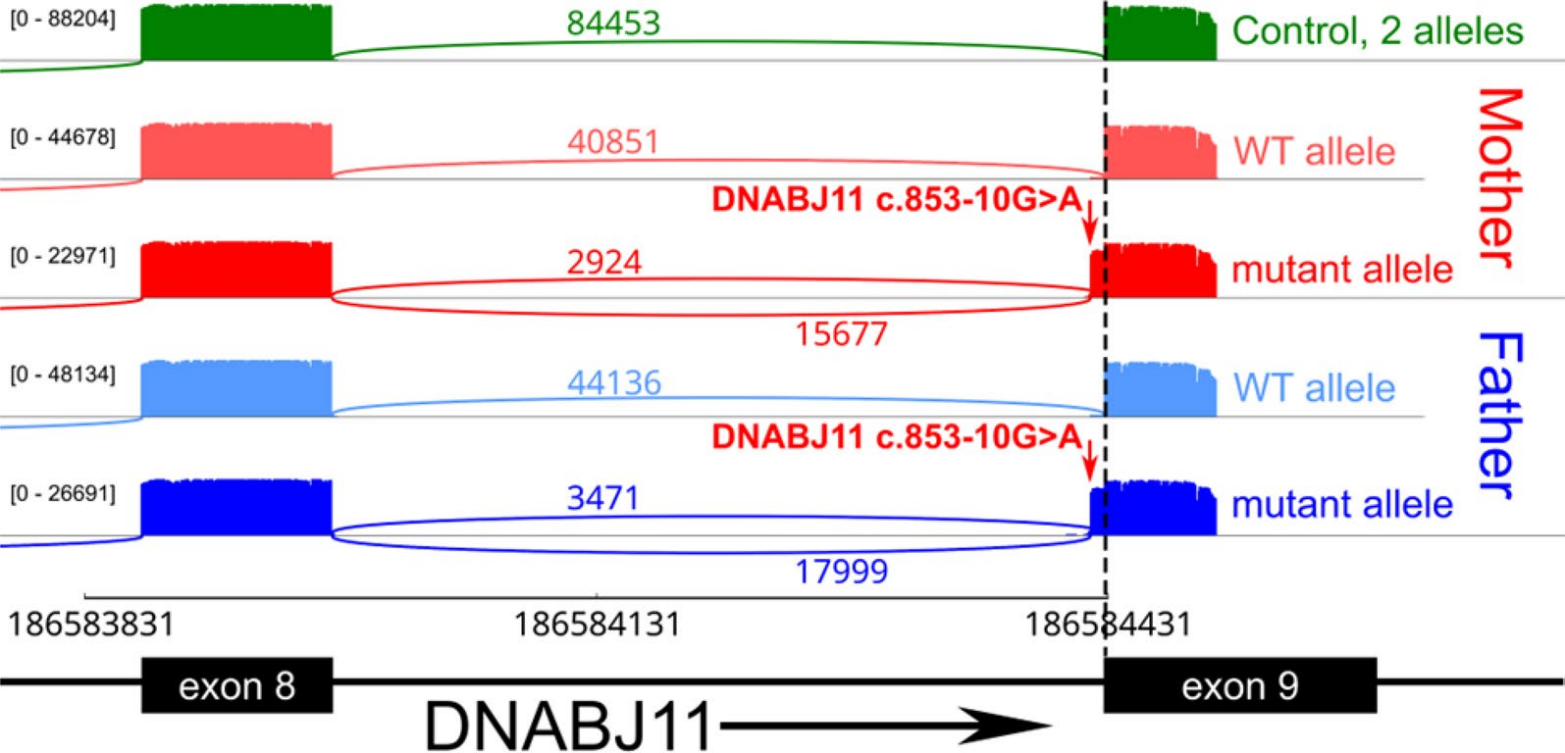
Sashimi plot of DNAJB11 c.853-10G > A splicing variant for interpretation of variant effect. The sashimi plot shows the splicing variant in the parents of the proband in PED002 (Mother = PED002A in red, Father = PED002B in blue) compared to an unrelated control adult (green). RT-PCR and nanopore sequencing demonstrates retention of 8 intronic bases (red arrows) of the 8th intron of DNAJB11 resulting from the identified variant on the mutant allele of both heterozygous parents

On re-review of the genomic data, an alternate gene in the ROH came under suspicion, *DNAJB11,* as it had recently been implicated in causing PKD [10, 22]. The *DNAJB11* variant in the ROH was a biparental homozygous splicing variant (NM_016306.6:c.853-10G > A), which was predicted to alter splicing by creation of an AG in the AG exclusion zone [27] (SpliceAI delta scores: AG: 0.98, AL: 0.31). As fetal samples were no longer available for RNA studies, RT-PCR in conjunction with Nanopore sequencing was performed on RNA from blood samples from the heterozygous parents. Mapped reads demonstrated activation of a cryptic acceptor site, leading to inclusion of the last 8 bases of intron 8 (Fig. 4). This results in a frameshift resulting in a premature termination codon 57 nucleotides from the last exon junction and loss-of-function. This evidence allowed this variant to be classified as pathogenic according to American College of Medical Genetics (ACMG) guidelines [28], resulting in diagnosis of ARPKD caused by a homozygous recessive *DNAJB11* pathogenic variant. The atypical renal cystic phenotype seen in the two fetuses in our family was also consistent with that seen in a previously reported individual with a homozygous splicing variant in *DNAJB11* (c.600-2A > C), in which irregular cystic proximal and distal tubules also occurred [22]. The overlap with our proband may suggest that a novel phenotype occurs commonly in fetal cases of PKD with biallelic *DNAJB11* variants and that this should be considered where this phenotype is observed.

Prior to these investigations, the run of homozygosity containing both the *TEME212* and the *DNAJB11* variant was returned in a research report to the family, who utilized pre-implantation testing for this homozygous region as a means for risk reduction. In addition, discovery of this pathogenic variant will allow the parents to be monitored for onset of PKD, as heterozygous *DNAJB11* mutation can also cause ADPKD later in life (average kidney failure onset beyond 60 years of age) [29]. The clinical team referred both parents for kidney and liver ultrasounds.

### Case 2: PED283

PED283 was a non-consanguineous family with a history of ADPKD. The mother, maternal uncle and maternal grandfather all had previous clinical diagnoses of ADPKD (Fig. 2B). The proband was a female fetus terminated at 27 + 5 weeks gestation due to large, echo-dense kidneys, empty renal pelvis and bladder, anhydramnios, ascites, small pericardial effusion and reduced cardiac contractility. At post-mortem analysis, both kidneys were confirmed to be grossly enlarged and edematous. Autolysis precluded definitive histological analysis, however cortico-medullary demarcation was absent from the cut surfaces, and numerous cystic structures were identified in the cortex and medulla (Fig. 3C). This was not however deemed to be typical for the family history of ADPKD due to the irregularity of the cysts and the prenatal onset. The cysts also showed extensive loss and detachment of their cuboidal epithelial lining, and beneath the capsule, there were areas of partially degraded immature blastemal tissue.

In the female proband, pathogenic compound heterozygous variants in the ADPKD gene *PKD1* were identified—a paternally inherited pathogenic missense variant (NM_001009944.3:c.9829C > T; p.Arg3277Cys) and a maternally inherited pathogenic stop-gain variant (NM_001009944.3:c.1597C > T; p.Gln533Ter). The paternally inherited variant has been reported *in trans* with terminating variants in previous cases of early-onset PKD and has been functionally shown in mice to be hypomorphic [5, 30], suggesting the father may be at reduced risk of developing significant ADPKD. Due to the original post-mortem diagnosis that this phenotype did not fit with the family history of ADPKD, and given the genetic finding, re-examination of the histopathology was undertaken. This identified multiple round to ovoid cysts throughout the renal parenchyma with glomerular involvement that lacked the characteristic elongated cysts typical of ARPKD. This suggested the two *PKD1* variants *in trans* caused a recessive PKD with an ADPKD-like cystic phenotype, which has previously been reported [19]. The *PKD1* variants were reported to the family, and screening was recommended for family members who may carry a pathogenic *PKD1* variant.

### Case 3: PED338

PED338 was a family with a clinical history of ADPKD. The father had renal cysts and reported that his father and siblings had a similar ADPKD phenotype (Fig. 2C). The female proband was terminated at 20 weeks gestation due to bilaterally enlarged symmetrical kidneys with multiple cysts of varying sizes. The family had also terminated a prior pregnancy due to bilateral cystic kidneys, but a post-mortem was not performed. At post-mortem, bilateral enlarged renal cysts were confirmed with diffuse cysts of approximately 1-10 mm in diameter (Fig. 3D). Abnormal biliary development in a mild ductal plate malformation in the liver was also identified. It was noted that renal microscopy was most suggestive of ADPKD but that the presence of abnormally biliary development in the liver could be supportive of ARPKD.

The female proband was homozygous for a biparental variant of uncertain significance in *PKD1* (NM_001009944.3:c.3494A > G; p.Asp1165Gly). A review of the histopathology was undertaken after genetic testing, which showed a thin and interrupted subcapsular nephrogenic zone with multiple round to ovoid cysts lined by flattened epithelium throughout the renal parenchyma. Cysts in the medullary region were generally larger than those under the capsule. Occasional residual glomeruli and small tubules were identified between the tubular cysts and were set within an edematous, mildly cellular stroma. Occasional small glomerular cysts were evident under the capsule and more deeply located. The cysts did not have the characteristic saccular elongated appearance running perpendicular to the capsule which is indicative of ARPKD; this is consistent with the finding of a homozygous variant in *PKD1*, which can cause an ADPKD-like cystic phenotype [19]. Hepatic changes resembling a ductal plate malformation was evident but observed in patchy distribution with some larger, more cystically dilated ducts evident. The *PKD1* variant was reported to the family, and a renal scan was also recommended for the mother, who was found to have two small cortical cysts.

### Case 4: PED138

PED138 was a consanguineous family with no reported history of PKD (Fig. 2D). The proband was a male fetus terminated at 24 + 5 weeks gestation due to large kidneys on ultrasound. At post-mortem, Potter sequence facies was observed, along with kidneys that appeared large with no recognizable corticomedullary differentiation. No cystic structures were detected, however kidney sections were severely autolyzed (Fig. 3E). It was also noted that the ureters were patent but hypoplastic, along with a small bladder. The final diagnosis was renal dysplasia.

The male proband was homozygous for a biparental pathogenic stop-gain variant in the ARPKD gene *PKHD1* (NC_000006.11:g.51921499G > A; c.1690C > T; p.Arg564Ter). This case was also referred for histological re-examination due to the original diagnosis of renal dysplasia. Kidney sections were again noted to be severely autolyzed but a reniform appearance was retained which is atypical for renal dysplasia. Cystic structures were scant or absent in some sections but an impression of mainly radial medullary collecting duct cysts typical for ARPKD were noted in other sections. No glomerular cysts were identified. This re-examination paired with the presence of a homozygous *PKHD1* variant confirmed the diagnosis of ARPKD.

### Case 5: PED297

PED297 was a non-consanguineous family with no reported history of PKD (Fig. 2E). The proband was a male fetus that was classified as a neonatal death at 33 weeks gestation. At 30 weeks, large echo-dense kidneys were identified along with severe oligohydramnios. At post-mortem, large multicystic kidneys were noted, with irregular rounded and elongated cysts in the medulla and inner cortex (Fig. 3F), along with a ductal plate malformation. No glomerular cysts were observed; however, it was noted that the cysts did not have the typical radial arrangement of ARPKD, and as such the findings were not typical for either ARPKD or ADPKD.

In the male proband, compound heterozygous variants in the ARPKD gene *PKHD1* were identified—a paternally inherited pathogenic stop-gain variant (NM_138694.4:c.11287C > T; p.Gln3763Ter) and a maternally inherited likely pathogenic deletion (NM_138694.4:c.9217_9231del; p.Trp3073_Trp3077del). The histopathology sections were again re-examined and whilst it was noted that there were some irregular and rounded cysts, there were also elongated cystic collecting ducts and tubules indicative of ARPKD.

## Discussion

Our results demonstrate the clinical utility of a genomic autopsy approach to fetal cases of PKD with atypical histology and/or family history, as summarized in Table 2. The five families presented here represent the majority of solved cases in our cohort that had polycystic kidneys as their only major phenotype identified at autopsy (13 total), likely due to the fact that the clinical team of families who had histology and family history congruent with typical PKD would have opted for a targeting sequencing panel over enrollment in the Genomic Autopsy Study. Though the cohort of 13 is small, our overall solve rate of 54% (5 atypical PKD, 2 other genetic disorders with a cystic renal phenotype) is in line with other studies of genomic testing in non-typical cystic renal disease [31]. In a 2021 study of pediatric and adult patients with monogenic kidney disorders where typical ADPKD had been excluded, 31 of 65 patients with a cystic kidney phenotype received a genetic diagnosis from whole exome sequencing [31].

**Table 2:** Summary of findings for genomic autopsy cases with polycystic kidney disease.

	Proband phenotype		Gene	Diagnosis	Variant(s)	Final classification (ACMG evidence)
	Typical for gene	Atypical for gene				
PED002	Enlarged kidneys, severe oligohydramnios	Irregular, jagged cystic tubules	*DNAJB11 *[25]	ARPKD	Biparental c.853-10G > A	Pathogenic (PVS1, PM2, PP1, PM3_Supporting, PP4)
PED283	Multiple round to ovoid cysts throughout renal parenchyma. Some glomerular cysts with glomerular remnants in walls	Prenatal onset of large, echo-dense kidneys. Some cysts irregular	*PKD1*	Biallelic *PKD1* PKD (ARPKD)	c.9829C > T; p.Arg3277Cys and c.1597C > T; p.Gln533Ter	Both pathogenic (Variant 1: PS1, PS3, PM3 Variant 2: PVS1, PM2, PM3)
PED338	Bilaterally enlarged kidneys. Bilateral enlarged renal cysts with diffuse haphazardly arranged cysts of approx. 1-10 mm in diameter	Prenatal onset, mild hepatic ductal plate malformation	*PKD1*	Biallelic *PKD1* PKD (ARPKD)	Biparental c.3494A > G; p.Asp1165Gly	Variant of uncertain significance (PM2, PP3_Moderate, PP4)
PED138	Enlarged kidneys. No corticomedullary definition, impression of radial medullary collecting duct cysts, no glomerular involvement	Cystic structures absent in some sections (severe autolysis)	*PKHD1 * [25]	ARPKD	Biparental c.1690C > T; p.Arg564Ter	Pathogenic (PVS1, PM2, PM3)
PED297	Enlarged, echo-dense kidneys. Elongated cystic collecting ducts and tubules. No glomerular involvement	Irregular rounded and elongated cystic structures	*PKHD1*	ARPKD	c.11287C > T; p.Gln3763 Ter and c.9217_9231del; p.Trp3073_Trp3077del	Pathogenic (PVS1, PM2, PP4) and likely pathogenic (PM2, PM4, PM3, PP4)

The phenotype, gene and variant/s, diagnosis and final classification (including ACMG evidence categories used) for each case detailed in this study. Where the case was previously included in the publication of the first 200 cases from the Genomic Autopsy study, this has been referenced (25). ACMG evidence classifications: PVS1: Very strong, null variant in a gene where loss of function is a known mechanism; PS1: Strong, same amino acid change as a previously established pathogenic variant, PS3: Strong, well-established supporting in vitro or in vivo functional studies; PM2: Moderate, absent or extremely low (recessive) in population data; PM3: Moderate, recessive and detected in trans with a pathogenic variant; PM4: Moderate, protein length changes as a result of in-frame indel or stop-loss variant; PP1: Supporting, cosegregation with disease in multiple affected family members in disease gene; PP3: Supporting, multiple lines of computational evidence support deleterious effect on gene; PP4: Supporting, patient’s phenotype or family history highly specific for monogenic disease.

The families presented here demonstrate that fetal phenotypes can be novel and/or variable, with histopathology findings not always fitting cleanly into either ARPKD or ADPKD. In post-mortem cases, histopathological diagnosis can be further complicated by the quality of the post-mortem sample, which can be highly variable. In PED138, autolysis severely hampered the histological examination. This impaired diagnosis, with the phenotype attributed to renal dysplasia. Through genomic autopsy, we were able to diagnose this proband with ARPKD and order a targeted re-examination of the histopathology, which further confirmed a phenotype suggestive of ARPKD.

In cases of fetal PKD, diagnosis can also be compounded by family history, particularly where a family history of ADPKD does not correlate with a fetal finding of severe early onset PKD. ADPKD is generally diagnosed as late-onset and therefore may be dismissed as a potential cause of fetal cases of PKD. There are however multiple reports in the literature of cases of severe early onset PKD phenotypes associated with autosomal recessive inheritance of classical ADPKD genes. A known mechanism of this is the inheritance of hypomorphic *PKD1* alleles, sometimes in *trans* with loss-of-function variants [19, 30, 32, 33]. In a recent study of very early onset PKD, 17% (5/30) had two hypomorphic *PKD1* variants, and 30% (9/30) carried a pathogenic variant on one allele and a likely hypomorphic variant on the other allele of the *PKD1* gene, demonstrating a clear mechanism for early onset ARPKD dues to biallelic *PKD1* variants [34]. Similarly, variants in *DNAJB11* were first reported to be associated with first ADPKD [10], and then later found to have a more severe phenotype if inherited in a recessive manner [22, 32]. As such, it is important that genes typically associated with ADPKD are not dismissed in cases of severe early-onset fetal PKD. This is true regardless of whether genomic sequencing is being conducted as part of a genomic autopsy, or as part of prenatal testing after large, hyperechogenic kidneys are detected on ultrasound. This is in line with the Kidney Disease Improving Global Outcomes (KDIGO) 2025 Clinical Practice guideline for the Evaluation, Management and Treatment of ADPKD, which recommend genetic testing for very early onset PKD, and recognize that ADPKD genes are also associated with recessive disease, even recommending carrier screening for partners of ADPKD patients who wish to start a family [8].

A finding of fetal PKD caused by autosomal recessive inheritance of classical ADPKD genes can also be of direct health benefit to the parents themselves. By conducting trio genomic analysis, we were able to confirm that in all three of our families where this inheritance pattern was seen (PED002, PED283 and PED338), both parents were heterozygous for a variant in a classical ADPKD gene, even though not all these parents had been previously diagnosed with ADPKD. A recommendation for further follow up testing was provided to the families’ clinical teams—we are aware of parents from PED002 and PED338 being referred for ultrasound, with small cysts found in the mother of PED338. A genetic diagnosis of an ADPKD-associated variant will allow the carriers to continue accessing kidney monitoring moving forwards.

Though a genomic autopsy added significant diagnostic value to the analysis of post-mortem cases of atypical PKD, it was not able to provide a diagnosis for all cases in the Genomic Autopsy Study cohort where polycystic kidneys were the only substantial finding. In addition, whilst tissue autolysis did not substantially impact DNA quality for genomic sequencing, in cases where the histology was atypical and/or impaired by autolysis, we were able to have the histology slides re-analyzed by a second pathologist to aid in variant interpretation—a service which may not be available in routine clinical practice. Finally, while sequencing costs were covered by research funding in this study, and trio whole genome sequencing is implemented as standard in many Australian national centers, the expense of trio whole genome sequencing may limit its accessibility in routine clinical care in some health services, and in other countries more broadly. Nevertheless, this work demonstrates the importance of interrogating both ARPKD and ADPKD genes in the setting of PKD, which could be achieved through a more accessible next-generation sequencing panel.

## Conclusions

As demonstrated by these families, genomic sequencing can add clarity to a post-mortem fetal PKD diagnosis where tissue autolysis or an atypical phenotype make diagnosis difficult. Additionally, if funding is available, a whole exome or whole genome approach can allow periodic or targeted re-analysis (e.g. PED002) and can also prevent causative variants being missed due to uncommon modes of inheritance, i.e. biallelic inheritance of an ADPKD gene causing severe disease. As such, if diagnostic costs require panel sequencing over whole genome/exome, it is important that classical ADPKD are included in the panel even where a diagnosis of ARPKD is suspected.

An accurate genetic diagnosis is of particular importance for families planning future pregnancies; an inherited recessive genetic finding carries a 25% risk of recurrence, and in the case of biallelic inheritance of an ADPKD gene, there is an additional 50% risk of the child inheriting a monoallelic ADPKD variant. A genetic diagnosis allows families to make informed reproductive decisions, and may also provide the potential for accessing IVF with pre-implantation genetic testing to assist families in their goal of having future healthy children, as seen with PED002. Additionally, findings can be of benefit to the parents’ health as well, as it may inform them if they are at risk of developing ADPKD in the future. As such, a genomic autopsy can be of benefit during post-mortem investigation of early-onset PKD, particularly in atypical presentations.

## Supplementary Information

Supplementary material 1.

Supplementary material 2.

## Data Availability

The genomic data generated in this study is not publicly available due to participant consent restrictions; however, controlled access to de-identified data may be requested in accordance with the procedures outlined in [25].
