# Supplementary material for: Atypical presentations of fetal polycystic kidney disease demonstrates the utility of a genomic autopsy for accurate post-mortem diagnoses
Source: Hum Genomics. 2025 Nov 12;19:132. doi: 10.1186/s40246-025-00844-4 (PMC12607061; doi:10.1186/s40246-025-00844-4)
Supplement: Supplementary file 1 — Supplementary material 1. [file 40246_2025_844_MOESM1_ESM.docx]

**Supplementary Table of Contents:**

Supplementary Methods

Bioinformatic analysis on NGS data

Supplementary Figure 1: IGV of maternal NM_001009944.3(PKD1):c.1597C>T variant in PED283.

Supplementary Figure 2: IGV of paternal NM_001009944.3(PKD1):c.9829C>T variant in PED283.

Supplementary Figure 3: IGV of biallelic NM_001009944.3(PKD1):c.3494A>G variant in PED338.

Dynamic Array for expression of TMEM212 in human tissue

Supplementary Table 1: Primer sequences for human tissue specific gene expression analysis

Tmem212 RNA *in-situ* hybridisation in mouse kidney

Tmem212 rt-PCR

Supplementary Table 2: Primer sequences for mouse kidney rt-PCR

Generation and characterisation of Tmem212 KO mice using CRISPR-Cas9 genome editing

Supplementary Figure 4: Tissue specific expression of TMEM212

Supplementary Figure 5: Tmem212 expression in the mouse embryonic kidney

Supplementary Figure 6: Kidney phenotype of wildtype and Tmem212 knockout mice

Supplementary Table 3: Evidence used to determine ACMG classification for each variant (Table included as excel file).

Supplementary References

**Supplementary Methods**

***Bioinformatic analysis on NGS data***

Primary and secondary analysis of sequencing data were performed as previously described in(1). Briefly, exomes and genomes were mapped to the human GRCh38 (hg38) genome assembly. SNPs and Indels were called according to GATK Best Practices; CNVs from exomes were called using default parameters for XHMM(2) and DECoN(3), while ROHs are detected from small variant VCFs using BCFtools. For genomes, CNVs were called using default parameters for Dragen’s Germline CNV Calling pipeline(4) while ROHs are detected as part of Dragen’s Small Variant Calling pipeline with the default value of 0.2 for the fraction of filtered calls (highest 10% and lowest 10% in DP values). Variants were curated, and analysed, using the in-house VariantGrid platform (https://github.com/SACGF/variantgrid) and the Emedgene (Illumina) software. Variants under all Mendelian inheritance models were considered, including autosomal dominant with incomplete penetrance (in asymptomatic parents). In addition to parent-proband trio analysis, a virtual panel analysis using proband data, was conducted using genes listed as green or amber in the Kidneyome_SuperPanel from PanelApp Australia; this facilitated the screening of digenic or oligenic inheritance involving two or more kidney genes. All homozygous variants flagged within the virtual panel analysis, were also manually inspected on IGV for potential runs of homozygosity and uniparental disomy. Pathogenicity assessment and subsequent variant classification was per recommendations from ACMG and ClinGen guidelines(5-7).

There was adequate sequencing coverage across the entire PKD1 locus in PED283 exome (IDT xGen Exome v2 capture) and PED338 genome. All three PKD1 variants were manually inspected on IGV and had adequate sequencing coverage (see Supplementary Figures 1-3).

*
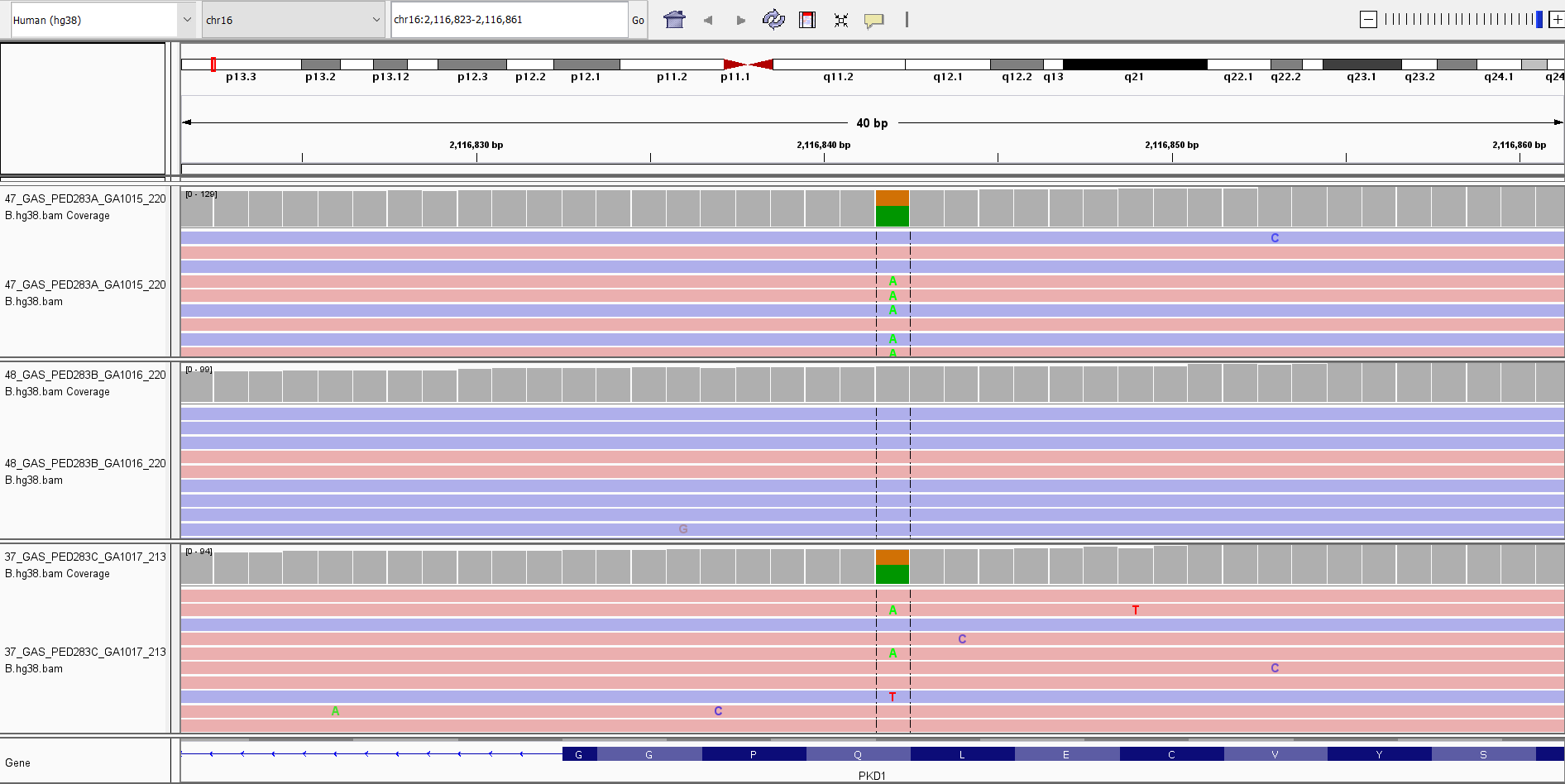
*


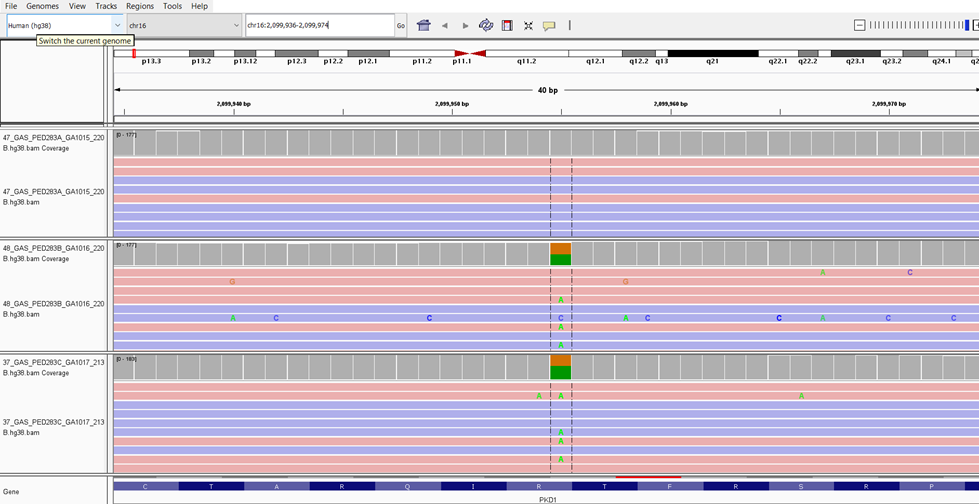


Proband

Father

Mother

***Supplementary Figure 1: IGV of maternal NM_001009944.3(PKD1):c.1597C>T variant in PED283 fetus. The heterozygous c.1597C>T stop-gain variant had an allele depth of 45 and a read depth of 78 in the proband.***

Proband

Father

Mother

***Supplementary Figure 2: IGV of paternal NM_001009944.3(PKD1):c.9829C>T variant in PED283. The heterozygous c.9829C>T missense variant had an allele depth of 88 and a read depth of 162 in the proband.***


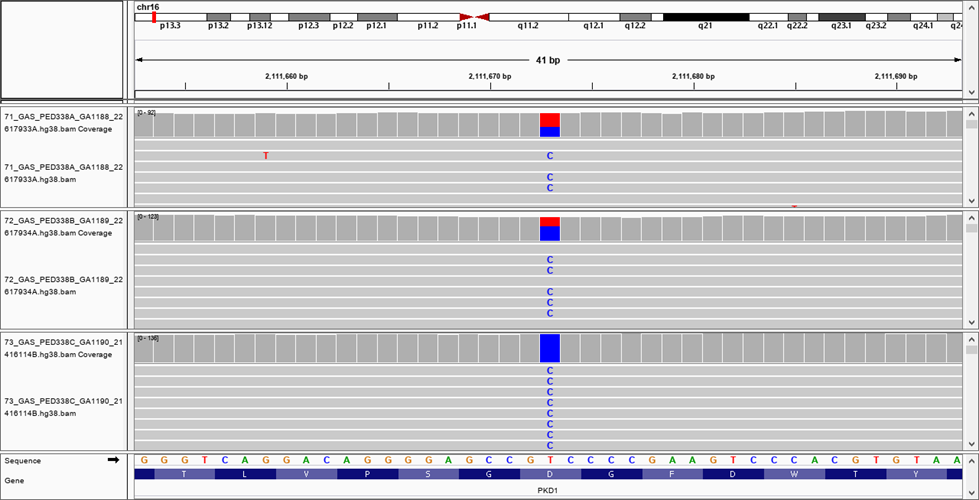


Mother

Father

Proband

***Supplementary Figure 3: IGV of biallelic NM_001009944.3(PKD1):c.3494A>G variant in PED338. The homozygous c. c.3494A>G missense variant had an allele depth of 129 in the proband.***

***Dynamic Array for expression of TMEM212 in human tissue***

Sample and assay mixes for the 96.96 Dynamic Array with EvaGreen (Fluidigm) were prepared according to the manufacturer’s protocol. Each assay and sample were added to their respective inlets on the ship, and loaded using the IFC controller software Load Mix (136x) script. The chips were then run on the BioMark HD using the recommended cycling parameters for a 96x96 chip on the Data Collection Software. Resutls were analysed using the Fluidign Real-Time PCR analysis software. Primer sequences listed in Table S1 below.

Table S1: Primer sequences for human tissue specific gene expression analysis

| **Gene** | **Forward Primer (5’ – 3’)** | **Reverse Primer (3’ – 5’)** |
| --- | --- | --- |
| TMEM212 | GCAATAGCCTTGGAATCTGC | AAGGAAAGGTAACTGCATAGCC |
| BM2 (housekeeping) | AATGTCGGATGGATGAAACC | TCTCTCTTTCTGGCCTGGAG |
| HPRT1 (housekeeping) | ACCCTTTCCAAATCCTCAGC | TCCTCCTCCTGAGCAGTCA |
| HMBS (housekeeping) | GTACCCACGCGAATCACTCT | AGCCTACTTTCCAAGCGGAG |
| RPLP0 (housekeeping) | GGATCTGCTGCATCTGCTTG | GCGACCTGGAAGTCCAACTA |

***Tmem212 RNA in-situ hybridisation in mouse kidney***

The in-situ probe sequence was amplified from pooled embryonic mouse kidney cDNA. The following primers were used: forward primer 5′-GTACACAGGATGGAGCGTTTG-3′, and reverse primer 5′-TGCCAGGGACACACAGAATA-3′. The 388bp amplicon was cloned into a pGEM-T Easy vector (Promega) and transformed into JM109 Competent Cells. Single colonies were cultured overnight, and clones were purified and absence of mutation was confirmed by sequencing. Plasmid DNA was purified by phenol-chloroform extraction, and used to synthesise antisense and sense RNA in-situ hybridisation probes.

Whole embryos from wildtype C57Bl/6 embryos were fixed in 4% paraformaldehyde and stored in 100% methanol. Whole mouse RNA in situ hydribisations were performed on the Intavis InsituPro Vsi. Tissue samples were rehydrated in decreasing concentrations of methanol/PBS-T and bleached with 6% H2O2, then permeabilsed with proteinase K. Hybridisation was performed overnight at 65°C with the digoxigenin-labeled probes. After washing, samples were blocked in 10% sheep srum, then incubated with anti-dioxigen antibody conjugated to alkaline phosphatase. Alkaline phosphatase activity was detected by incubating samples in a staining solution, with images taken using the SZX10 research stereomicroscope (Olympus). Images processed with OpenLab 2.2. software.

***Tmem212 rt-PCR***

Kidneys were dissected from Wildtype C57Bl/6 mice embryos at E14.5 – E17.5, and RNA was extracted using an RNeasy MiniKit (Qiagen). cDNA was generated using the SuperScript III Reverse Transcriptase kit (Invitrogen), and rt-PCR was performed in the LightCycler 480 II system (Roche) using the SYBR Green I Mater protocol. Primer sequences used for rt-PCR in Table S2 below.

Table S2: Primer sequences for mouse kidney rt-PCR

| **Gene** | **Forward Primer (5’ – 3’)** | **Reverse Primer (3’ – 5’)** |
| --- | --- | --- |
| Tmem212 | GATGGAGCGTTTGGATTGCC | TACGAAGGTGAACACGGCTT |
| B2m (housekeeping) | GGTGACCCTGGTCTTTCTGG | TGTTCGGCTTCCCATTCTCC |
| Hprt1 (housekeeping) | GATCAGTCAACGGGGGACAT | GGGGCTGTACTGCTTAACCA |
| Hmbs (housekeeping) | ATTCAGTGCCATCGTCCTGG | GATATCCTGGTCCTTGGCTCG |
| Rplp0 (housekeeping) | GGACCTCACTGAGATTCGGG | AGCCTGGAAGAAGGAGGTCT |

***Generation and characterisation of Tmem212 KO mice using CRISPR-Cas9 genome editing.***

CRISPR guides were designed to target exon 1 of mouse Tmem212; guide sequence was 5’-GGTACACAGGATGGAGCGTT - 3’. Tmem212 KO mice were generated at the South Australian Genome Editing Facility by injecting C57Bl/6 zygotes cytoplasmically with CRIPSR reagents, and then transferring these zygotes into pseudo-pregnant recipient mice. Founder pups were screened for indels leading to frameshifts and premature stop codons, and 3 mouse lines were generated. Mice were humanely killed at various time points, and kidneys assessed by histology.


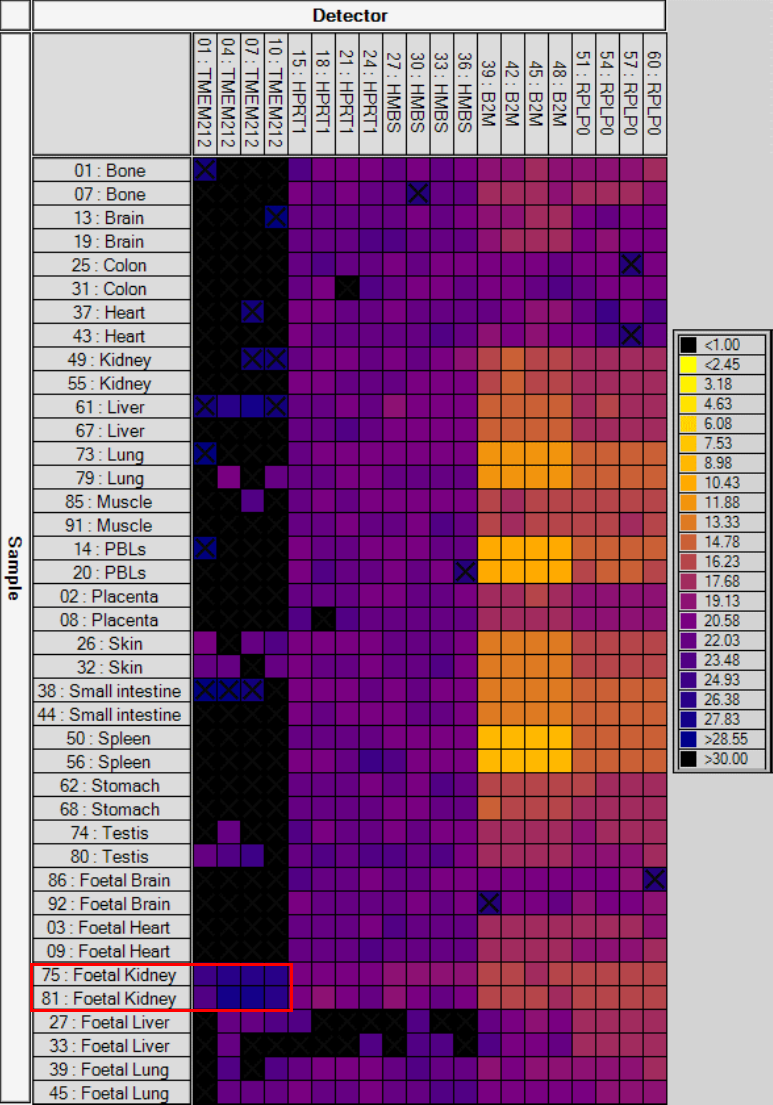


***Supplementary Figure 4: Tissue specific expression of TMEM212. A gene expression dynamic array was performed on twenty human tissues, and showed that TMEM212 is expressed in fetal kidney (highlighted in red). HPRT1, HMBS, B2M and RPLP0 were used as housekeeping genes.***


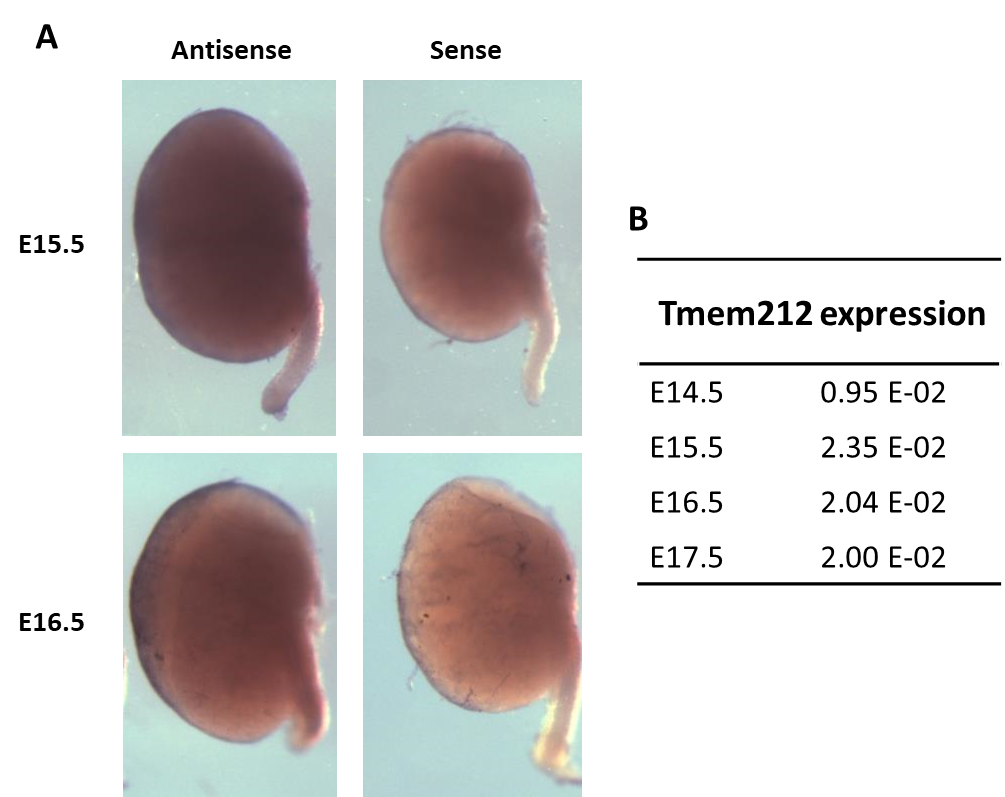


***Supplementary Figure 5: Tmem212 expression in the mouse embryonic kidney. A) Whole mount RNA in situ hybridisation for Tmem212 expression in the mouse kidney at E15.5-E16.5 (magnified 3.2x). The antisense probe showed expression of Tmem212 in the fetal mouse kidney at this time point. The sense probe was used as a negative control, and showed no expression. B) rt-PCR performed in parallel with the RNA in situ hybridisation also shows expression of Tmem212 in the developing kidney at E15.5-E16.5.***


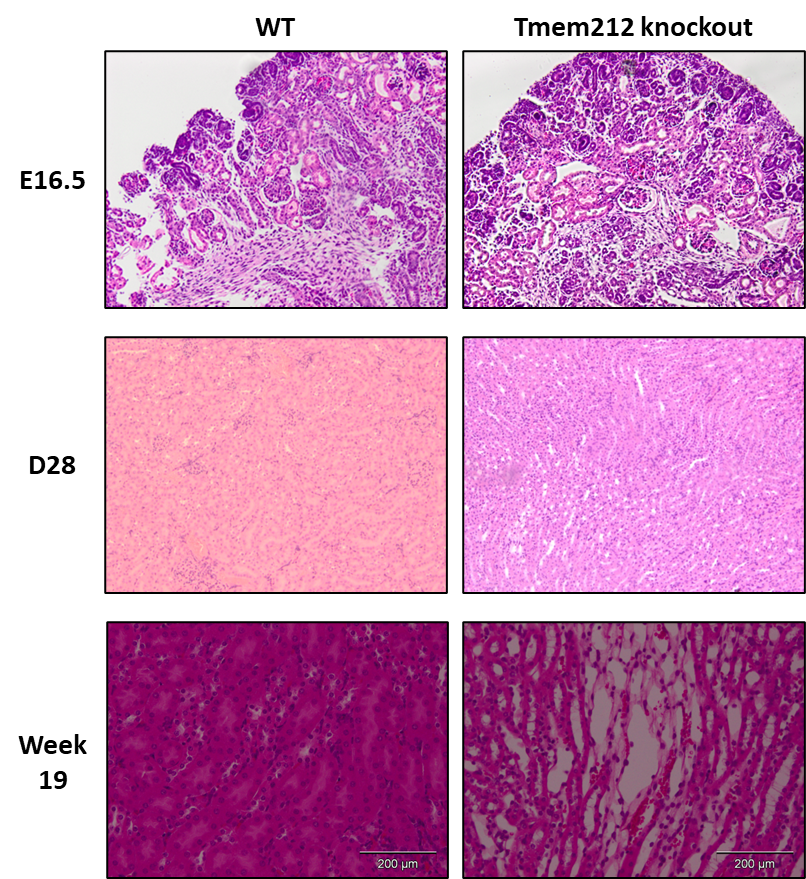


***Supplementary Figure 6: Kidney phenotype of wildtype and Tmem212 knockout mice. Kidneys from wildtype and Tmem212 knockout mice was assessed by histology (haematoxylin and eosin statin) at embryonic day 16.5, 28 days after birth, and 19 weeks after birth. E16.5 images at 25x magnification, D28 images at 10x magnification, and week 19 images at 100x magnification.***

***Supplementary Table 3: Evidence used to determine ACMG classification for each variant.***

***The evidence used for each reported variant to assign ACMG categories.***

**Supplementary References**

1. Byrne AB, Arts P, Ha TT, Kassahn KS, Pais LS, O'Donnell-Luria A; Broad Institute Center for Mendelian Genomics; Babic M, Frank MSB, Feng J, Wang P, Lawrence DM, Eshraghi L, Arriola L, Toubia J, Nguyen H; Genomic Autopsy Study Research Network; McGillivray G, Pinner J, McKenzie F, Morrow R, Lipsett J, Manton N, Khong TY, Moore L, Liebelt JE, Schreiber AW, King-Smith SL, Hardy TSE, Jackson MR, Barnett CP, Scott HS. Genomic autopsy to identify underlying causes of pregnancy loss and perinatal death. Nat Med. 2023;29(1).

2. Fromer M, Purcell, S.M. Using XHMM Software to Detect Copy Number Variation in Whole-Exome Sequencing Data. Curr Protoc Hum Genet. 2014;81.

3. Fowler A. DECoN: A Detection and Visualization Tool for Exonic Copy Number Variants. Methods Mol Biol. 2022;2493:77-88.

4. Behera S, Catreux S, Rossi M, Truong S, Huang Z, Ruehle M, et al. Comprehensive genome analysis and variant detection at scale using DRAGEN. Nature Biotechnology. 2025;43(7):1177-91.

5. Riggs ER, Andersen EF, Cherry AM, Kantarci S, Kearney H, Patel A, et al. Technical standards for the interpretation and reporting of constitutional copy-number variants: a joint consensus recommendation of the American College of Medical Genetics and Genomics (ACMG) and the Clinical Genome Resource (ClinGen). Genet Med. 2020;22(2):245-57.

6. Rehm HL, Berg JS, Brooks LD, Bustamante CD, Evans JP, Landrum MJ, Ledbetter DH, Maglott DR, Martin CL, Nussbaum RL, Plon SE, Ramos EM, Sherry ST, Watson MS,. ClinGen The Clinical Genome Resource. N Engl J Med. 2015;372:2235-42.

7. Harrison SM, Biesecker LG, Rehm HL. Overview of Specifications to the ACMG/AMP Variant Interpretation Guidelines. Current Protocols in Human Genetics. 2019;103(1):e93.
